# Epigenomic Remodeling by Lipid Metabolism: A Cross-Disease Perspective on Cancer and Brain Disorders

**DOI:** 10.3390/genes17091134

**Published:** 2026-09-17

**Authors:** Asma Rafique, Adeel Farooq, Clara E. Cho, Marica Bakovic

**Affiliations:** 1 Department of Human Health Sciences, College of Biological Science, University of Guelph, Guelph, ON N1G 2W1, Canada; arafique@uoguelph.ca (A.R.); claracho@uoguelph.ca (C.E.C.); 2 Department of Food Science, Ontario Agricultural College, University of Guelph, Guelph, ON N1G 2W1, Canada; afaroo04@uoguelph.ca

**Keywords:** lipid metabolism, epigenomics, acetyl-CoA, DNA methylation, histone acetylation, cancer metabolism, neurodegeneration, metabolic–epigenetic crosstalk

## Abstract

Lipid metabolism is no longer viewed simply as a source of membrane biomass or ATP. It is now proposed as a regulatory system that determines which metabolites reach the nucleus, which chromatin-modifying enzymes are engaged, and which transcriptional states become stable during disease progression. This review develops a mechanistic cross-disease framework emphasizing metabolite flux, subcellular compartmentalization, cell-type specificity, and the temporal persistence of chromatin states. We synthesize recent evidence linking lipid metabolism to chromatin remodeling in cancer, Alzheimer’s disease, and Parkinson’s disease, and we highlight how emerging single-cell multi-omics and spatial lipidomics may shape potential metabolism-targeted epigenetic therapies. In both cancer and brain disorders, lipid flux alters the availability of acetyl-CoA, S-adenosylmethionine, NAD+, and bioactive fatty acid derivatives, thereby reshaping histone acetylation, DNA and histone methylation, chromatin accessibility, and transcription-factor activity. In cancer, de novo lipogenesis, acetate scavenging, fatty acid oxidation, and phospholipid remodeling sustain nuclear acetyl-CoA pools, promote oncogenic super-enhancers, and support survival under hypoxia, acidosis, and therapy stress. In neurodegenerative disease, mitochondrial dysfunction, cholesterol dyshomeostasis, sphingolipid imbalance, oxysterol accumulation, and microglial lipid droplet formation may distort the epigenetic programs required for neuronal maintenance, synaptic plasticity, and inflammatory resolution. However, neurodegenerative disorders are not uniformly hypoacetylated; instead, they exhibit stage-, region- and cell-type-specific chromatin remodeling, with hypoacetylation at some vulnerable loci coexisting with hyperacetylation at others. By comparing lipid–epigenome coupling across proliferative and degenerative conditions, we argue that the same metabolic nodes can yield markedly different pathological outcomes depending on cellular context, providing a basis for more precise therapeutic design.

## 1. Introduction

The epigenome provides the functional layer through which genetically identical cells acquire distinct phenotypes, maintain lineage identity, and adapt to environmental stress [1,2]. Among the epigenetic mechanisms, DNA methylation, histone modifications, and chromatin accessibility do not act in isolation; rather, they are closely influenced by cellular metabolic pathways. Many chromatin enzymes depend directly on metabolite availability, which means that the state of lipid, glucose, and one-carbon metabolism can determine whether particular genomic loci remain transcriptionally permissive or silenced [3].

Within this framework, lipid metabolism occupies a uniquely important position because it links membrane biology, organelle function, redox balance, and carbon storage to chromatin regulation [4]. Lipid pathways generate acetyl-CoA for histone acetylation, influence methionine-cycle flux and S-adenosylmethionine availability through choline and phosphatidylcholine metabolism, alter NAD+-dependent deacetylation through mitochondrial redox state, and signaling lipids and short-chain fatty acids that modulate nuclear receptors, histone deacetylases, and inflammatory transcriptional programs. In other words, lipid metabolism is not merely a downstream consequence of gene regulation but a fundamental determinant of chromatin state, regulatory architecture, and cellular fate [4].

The relevance of the lipid–epigenome axis is especially evident in cancer [5]. Tumors sustain growth under hypoxia, nutrient limitation, acidic conditions, and therapeutic stress by reprogramming de novo lipogenesis, fatty acid uptake, β-oxidation, acetate utilization, and phospholipid turnover. These changes do more than support membrane synthesis [5]. They also channel carbon units into nuclear acetyl-CoA pools, stabilize histone acetylation at oncogenic enhancers, shape transcription-factor binding, and influence chromatin programs that govern stemness, immune escape, ferroptosis sensitivity, and metastatic competence [6,7]. Recent work has made clear that the cancer epigenome responds not only to total metabolite abundance but to pathway routing and compartmentalization, such as whether acetyl units are generated by ACLY, ACSS2, or nuclear pyruvate dehydrogenase under specific microenvironmental constraints [8].

Another area where the lipid–epigenome axis makes a compelling picture is in brain disorders [9,10]. The brain is exceptionally rich in lipids, yet neurons, astrocytes, oligodendrocytes, and microglia oversee lipid synthesis, transport, oxidation, and recycling in profoundly different ways. Neurodegenerative diseases, therefore, reveal a cell-type-specific version of lipid–epigenome coupling. This coupling is also disease-stage-dependent; a neuronal population may lose acetylation at plasticity-related loci in one phase, whereas reactive microglia or astrocytes may acquire hyperacetylated inflammatory or lipid-handling enhancers in another phase [9,10]. In Alzheimer’s disease (AD), disturbances in cholesterol handling, oxysterol balance, astrocyte reactivity, and microglial lipid storage are increasingly linked to altered chromatin states that favor inflammatory persistence and synaptic dysfunction [11]. In Parkinson’s disease (PD), changes in sphingolipid metabolism, mitochondrial redox homeostasis, and DNA methylation or hydroxymethylation intersect with dopaminergic vulnerability and maladaptive glial responses [10].

Despite rapid progress, most reviews still discuss cancer metabolism and neurodegeneration separately. A deeper cross-disease perspective is needed because similar metabolic nodes can generate opposite phenotypes depending on cellular context; surplus acetyl-CoA may stabilize oncogenic plasticity, whereas compartmental failure of acetyl-CoA production may lock neurons or glia into degenerative states. In this review, we therefore emphasize four mechanistic principles that cut across diseases: metabolite flux rather than static abundance, subcellular compartmentalization of metabolite production, cell-type specificity of lipid handling, and the persistence of chromatin states. This framework helps explain why common lipid intermediates can drive proliferation in one setting and vulnerability in another, while also revealing therapeutic opportunities at the interface of metabolism and epigenetic regulation [8,9].

### Literature Search Strategy and Scope

This article is a narrative review, not a systematic review. To improve transparency and reproducibility, we identified and updated the literature through targeted, iterative PubMed/MEDLINE searches, supplemented by manual checks of reference lists and citation links, with the search updated through 10 September 2026. Free-text terms and relevant MeSH concepts were combined across three domains: lipid metabolism (e.g., “lipid metabolism,” fatty acids, cholesterol, sphingolipids, phospholipids, acetyl-CoA, acetate, ACLY, ACSS2, choline, phosphatidylcholine, and one-carbon metabolism), epigenetic regulation (e.g., epigenetics/epigenomics, chromatin, histone acetylation, DNA/histone methylation, crotonylation, lactylation, and beta-hydroxybutyrylation), and disease context (cancer/neoplasms and neurodegenerative disorders including AD, PD, HD, ALS, and MS). Corresponding MeSH terms included “Lipid Metabolism,” “Epigenesis, Genetic,” “Histones,” “DNA Methylation,” “Neoplasms,” and “Neurodegenerative Diseases.” No lower publication-date restriction was imposed because foundational studies were retained for the mechanisms they established, which are still used in current models.

Priority was given to peer-reviewed original studies that directly connected a lipid pathway, metabolite, or lipid-handling process to a chromatin or epigenetic readout, and to recent high-quality reviews used to frame broader mechanisms. Association-only lipidomic or epigenomic studies were included when they provided disease or cell-type context but were not treated as equivalent to perturbation-based mechanistic evidence. Evidence from genetic or pharmacologic perturbation, rescue experiments, isotope tracing, locus-specific chromatin assays, and integrated multi-omics was weighted more strongly when evaluating causality. Contradictory findings were retained and interpreted in relation to cell type, disease stage, nutrient state, and subcellular compartment rather than forced into a single directional model. AI-assisted tools, where used for language organization, were not treated as literature databases or evidence sources; scientific interpretation and bibliographic details were checked against the original publications.

## 2. Fundamental Mechanisms of Lipid–Epigenome Crosstalk

**Figure 1:**
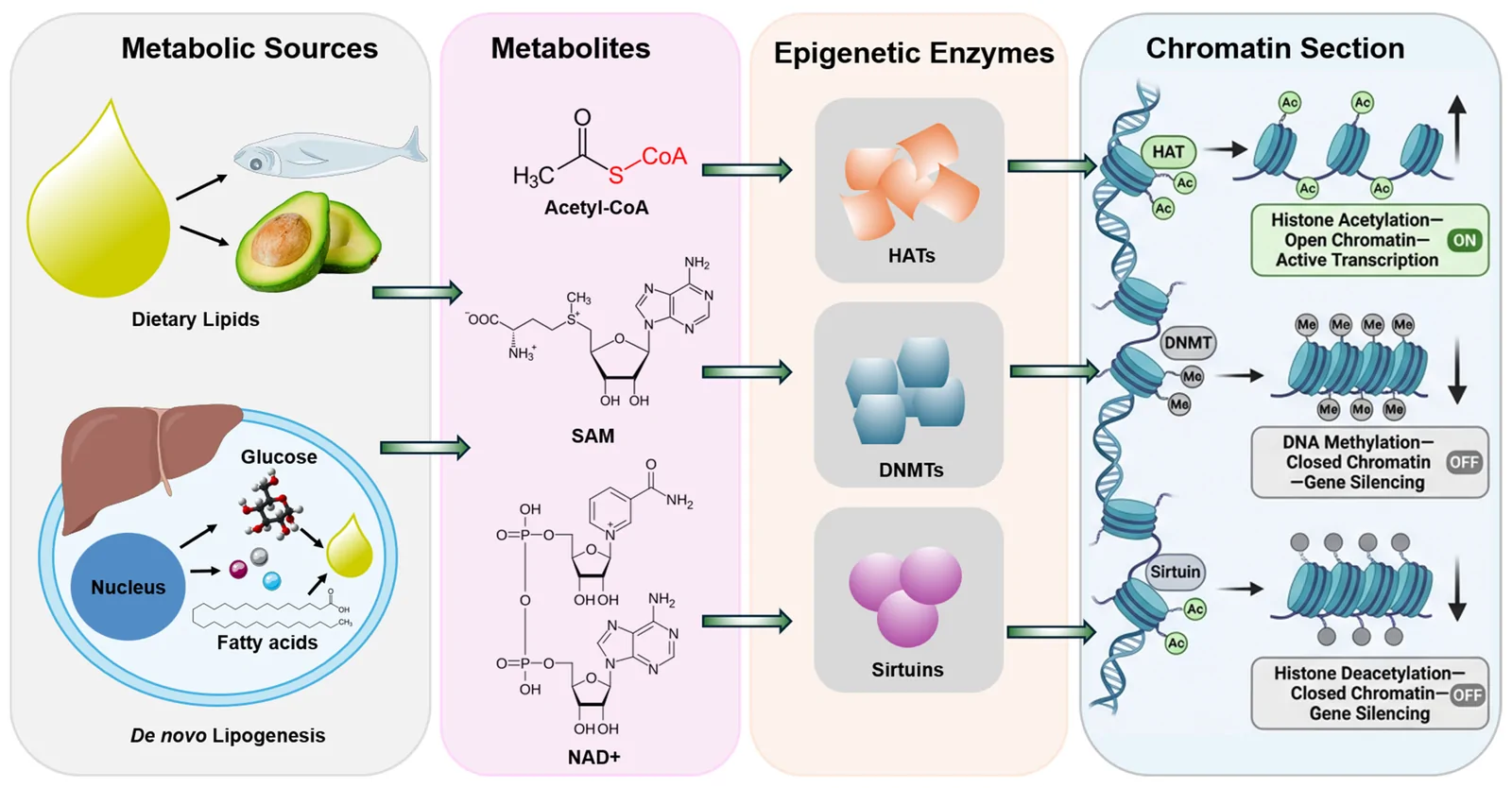
**The Metabolic-Epigenetic Axis: Lipid Metabolism to Gene Expression.** Schematic representation of how lipid metabolism generates various metabolites (e.g., Acetyl-CoA, SAM, NAD+, fatty acids) that serve as substrates or cofactors for epigenetic enzymes (e.g., HATs, HDACs, DNMTs, HMTs). These enzymes, in turn, modify the epigenome (DNA methylation, histone modifications), regulating gene expression and determining cellular phenotype. Feedback loops exist where gene expression regulates metabolic enzymes, and the epigenome can influence metabolic pathways. In this schematic, metabolite labels indicate the chromatin-accessible cytosolic/nuclear pools and enzyme-local substrate availability rather than total whole-cell abundance; therefore, acetyl-CoA, SAM, and NAD+ should be interpreted as regulatory pools capable of influencing chromatin-modifying enzymes.

The intricate relationship between lipid metabolism and epigenomic regulation is mediated by several key metabolic intermediates that serve as substrates or cofactors for epigenetic enzymes [2]. These metabolites link the cell’s metabolic state to its gene expression profile, enabling a dynamic response to internal and external cues (Figure 1).

This crosstalk should be viewed as a problem of nuclear metabolite access rather than simple whole-cell metabolite abundance. Chromatin-modifying enzymes respond dynamically to substrate availability, cellular redox status, and locus-specific regulatory factors [3]. Accordingly, disease states are often driven by changes in how metabolites are routed between mitochondria, cytosol, lysosomes, lipid droplets, and the nucleus [4]. This emphasis on flux and compartmentalization is particularly important for lipid biology because fatty acid synthesis, beta-oxidation, phospholipid turnover, and cholesterol handling occur across multiple organelles and cell types.

Throughout this review, flux, pool size, and compartmentalization are distinct concepts. Flux denotes the rate at which atoms move through a pathway; pool size denotes the amount or concentration measured at a given time; and compartmentalization denotes the spatial restriction of metabolites and enzymes to organelles or local nuclear microenvironments. A large pool can coexist with low flux, whereas a small pool can turn over rapidly. Flux is commonly estimated by stable-isotope tracing coupled to mass spectrometry and kinetic modeling, whereas compartment-resolved measurements require rapid fractionation or sensor-based approaches. Methods such as stable isotope labeling of essential nutrients in cell culture-subcellular fractionation (SILEC-SF) use isotope-labeled internal standards during fractionation to quantify acyl-CoA thioesters and have demonstrated that the nuclear acyl-CoA profile differs from that of the cytosol [12]. Nevertheless, exact free metabolite concentrations in the nucleus remain technically difficult to define because fractionation can permit leakage or exchange and because chromatin enzymes may respond to local enzyme-proximal pools rather than to a uniform nuclear concentration [12,13]. Thus, we use “nuclear availability” to mean an operationally accessible local pool, not a single universal nuclear concentration.

Metabolite abundance also does not by itself determine which genomic loci acquire an epigenetic mark. Locus specificity is determined by the chromatin regulatory machinery. Sequence-specific transcription factors recruit epigenetic “writers,” including p300/CBP, DNA methyltransferases (DNMTs), and histone methyltransferases (HMTs), whereas “erasers,” such as histone deacetylases (HDACs) and histone demethylases, remove these modifications. “Readers,” including bromodomain-containing proteins, recognize these epigenetic marks and recruit additional regulatory complexes that stabilize transcriptional programs and downstream regulatory states [13,14]. Pre-existing accessibility and enhancer architecture further constrain where these complexes act. Metabolic enzymes can add another layer of local control. Under glucose deprivation, for example, phosphorylated ACSS2 enters the nucleus, associates with TFEB at lysosomal and autophagy gene promoters, and locally generates acetyl-CoA that supports histone H3 acetylation and transcription [15]. These data provide a mechanistic route from a metabolic change to locus-specific chromatin regulation, but comparable targeting mechanisms remain incompletely defined for many lipid-sensitive marks and disease contexts [13].

### 2.1. Acetyl-CoA and Histone Acetylation

Recent work has refined that acetyl-CoA is best understood as a compartmentalized chromatin substrate [7]. Mitochondrial acetyl-CoA cannot freely diffuse into the nucleus; thus, cells rely on citrate export followed by ATP-citrate lyase (ACLY), acetate capture by acetyl-CoA synthetase 2 (ACSS2), and, in some contexts, nuclear pyruvate dehydrogenase activity to generate local acetyl donors. This nuclear supply is important because promoter acetylation, enhancer acetylation, and super-enhancer maintenance respond to local acetyl-CoA availability [16]. Lipid synthesis and lipid oxidation influence chromatin not simply by increasing total cellular acetyl-CoA, but by determining whether acetyl units are routed toward fatty-acid synthesis, mitochondrial oxidation, or nuclear histone acetylation [16,17,18].

Acetyl-CoA is the sole donor of acetyl groups for histone acetyltransferases (HATs), enzymes that catalyze the acetylation of lysine residues on histone tails [7], thus situated at the intersection of metabolism and epigenetic modification. Histone acetylation generally leads to a more open chromatin structure, facilitating the binding of transcription factors and promoting gene expression. Conversely, histone deacetylases (HDACs) remove these acetyl groups, leading to chromatin condensation and transcriptional repression. The cellular availability of acetyl-CoA directly influences the activity of HATs, thereby modulating global and locus-specific gene expression [16,17].

Acetyl-CoA can be generated from various metabolic pathways. In the cytoplasm, it is primarily produced from citrate by ACLY, linking glucose and fatty acid metabolism to acetyl-CoA pools [6,7]. Fatty acid oxidation (FAO) generates acetyl-CoA inside mitochondria; mitochondrial acetyl-CoA itself does not freely cross the inner mitochondrial membrane, so its carbon can influence cytosolic and nuclear acetyl-CoA pools only indirectly through exportable intermediates and shuttle pathways, depending on cellular state [7]. In certain contexts, such as hypoxic tumor microenvironments, nuclear ACSS2 converts acetate to acetyl-CoA, providing a localized acetyl donor for histone acetylation [15,18]. This direct link underscores how alterations in lipid synthesis and breakdown can impact the epigenome through the routing and localization of acetyl-CoA rather than through whole-cell abundance alone [16,18].

### 2.2. S-Adenosylmethionine (SAM) and Methylation

S-adenosylmethionine (SAM) is another crucial metabolic intermediate that serves as the universal methyl donor for DNA methyltransferases (DNMTs) and histone methyltransferases (HMTs) [15]. DNMTs catalyze the addition of a methyl group to cytosine residues, primarily in CpG dinucleotides, leading to DNA methylation, which is generally associated with gene silencing [19]. HMTs, on the other hand, methylate lysine and arginine residues on histones, with the functional outcome (activation or repression) depending on the specific residue and degree of methylation [19].

Choline and phosphatidylcholine are intimately linked to methylation and provide a biochemical bridge between membrane lipid metabolism and epigenetic modification [20,21]. Choline may be incorporated into phosphatidylcholine through the Kennedy pathway, oxidized to betaine to donate methyl groups to homocysteine, or recycled during phospholipid remodeling [20,22]. These competing fates determine methionine-cycle flux and the availability of SAM. Thus, high membrane demand in proliferating cancer cells, or defective phospholipid turnover in neural tissue, can alter DNA and histone methylation capacity through changes in choline allocation and SAM/SAH balance [4,23].

The synthesis of SAM is closely tied to the one-carbon metabolism pathway, which is influenced by various nutrients, including folate, vitamin B12, and choline [23,24]. While not directly a lipid, choline metabolism can impact SAM levels. For instance, the oxidation of choline to betaine contributes to the methionine cycle, thereby influencing SAM production [21]. Extending this, dysregulation in pathways related to lipid precursors may also affect DNA and histone methylation patterns.

### 2.3. NAD+ and Sirtuins

Nicotinamide adenine dinucleotide (NAD+) is a coenzyme critical for numerous metabolic reactions, including those involved in lipid metabolism [25,26]. It is also an essential cofactor for sirtuins (SIRT1-7), a class of NAD+-dependent deacetylases that function as HDACs [25,27]. Sirtuins play a vital role in regulating gene expression, metabolism, and aging by removing acetyl groups from histones and other non-histone proteins. The activity of sirtuins is directly coupled to the cellular NAD+/NADH ratio, meaning that metabolic states influencing this ratio (e.g., changes in fatty acid oxidation or glycolysis) can modulate sirtuin activity and, consequently, the epigenome [28]. For example, increased FAO can elevate NAD+ levels, thereby activating sirtuins and promoting deacetylation [25,26].

NAD+-linked chromatin control also connects lipid metabolism to stress adaptation. Each cycle of mitochondrial beta-oxidation reduces NAD+ to NADH and FAD to FADH2; efficient electron transport then reoxidizes NADH to NAD+, sustaining sirtuin-dependent deacetylation [25,27]. By contrast, hypoxia, electron-transport-chain damage, or mitochondrial overload produces NADH accumulation and a lower NAD+/NADH ratio, thereby limiting SIRT1-SIRT7 activity and changing histone and non-histone deacetylation [25]. Ketone-body metabolism adds a second layer; beta-hydroxybutyrate oxidation consumes NAD+ and produces NADH, whereas beta-hydroxybutyrate can also directly inhibit class I/II HDACs and contribute to noncanonical lysine beta-hydroxybutyrylation marks [29]. These mechanisms explain why the direction of the FAO-sirtuin relationship depends on respiratory pathways rather than FAO rate alone. These redox effects modify sirtuin activity, especially under fasting, hypoxia, or energetic stress [29]. In addition to these redox-dependent effects, beta-hydroxybutyrate and some short-chain fatty acids can influence chromatin directly through HDAC inhibition or alternative acylation marks, as discussed below [30]. These observations expand beyond acetylation and methylation alone, suggesting that lipid-responsive chromatin states may be encoded through multiple acyl modifications and deacylase pathways.

### 2.4. Other Lipid-Derived Metabolites

Beyond these central players, other lipid-derived metabolites and pathways also contribute to epigenomic remodeling:

#### 2.4.1. Fatty Acids and Nuclear Receptors

Specific fatty acids, such as butyrate produced by gut microbiota, can directly inhibit HDACs, lead to hyper-acetylation and altered gene expression [31,32]. Long-chain fatty acids, eicosanoids, oxysterols, and bile acids also function as ligands or modulators for lipid-sensing nuclear receptors, including PPARs, LXRs, FXR, and RXR [33,34,35]. These receptors relay lipid abundance into transcriptional output by binding regulatory DNA elements and recruiting coactivators or corepressors with HAT, HDAC, and methyltransferase activities [33,36]. Nuclear receptor signaling is therefore an epigenetic mechanism as well as a metabolic sensor, especially in cancer, inflammatory glia, and gut–liver–brain communication.

#### 2.4.2. Sphingolipids

These complex lipids are integral components of cell membranes and signaling molecules [37,38]. Dysregulation of sphingolipid metabolism has been linked to changes in DNA methylation and histone modifications, particularly in neurodegenerative diseases [39].

#### 2.4.3. Cholesterol

Cholesterol and its metabolites can influence epigenetic marks, with studies showing links between cholesterol biosynthesis pathways and histone acetylation in the brain [40,41,42]. A direct biochemical connection arises because acetyl-CoA is the first carbon precursor committed to the cholesterol/mevalonate pathway: two acetyl-CoA molecules form acetoacetyl-CoA, a third acetyl-CoA generates HMG-CoA, and HMG-CoA reductase then produces mevalonate [43]. This creates competition and coordination between lipid-anabolic flux and acetyl-CoA availability for histone acetylation. In neurons, this relationship has been demonstrated functionally, that astrocytic ApoE reprograms neuronal cholesterol metabolism and H3K27ac-dependent gene expression, linking neuronal cholesterol handling to histone-acetylation-mediated outcomes [40].

These diverse mechanisms highlight the multifaceted ways in which lipid metabolism can sculpt the epigenomic landscape, ultimately impacting cellular function and disease progression [41,44].

### 2.5. Emerging Lipid-Responsive Epigenetic Modifications

Although histone acetylation and DNA methylation remain the most extensively studied mechanisms linking lipid metabolism to gene regulation, recent discoveries have substantially expanded the repertoire of metabolite-sensitive chromatin modifications [45,46]. Advances in mass spectrometry and chromatin biology have shown that acyl-CoA derivatives generated during lipid, ketone-body, and intermediary metabolism can directly modify histone lysine residues [13,47]. These modifications function as metabolic sensors, enabling chromatin to respond dynamically to nutrient availability, redox state, mitochondrial activity, and lipid flux. Importantly, many of these modifications have been implicated in cancer progression, neurodegeneration, aging, inflammation, and metabolic disease.

#### 2.5.1. Histone Beta-Hydroxybutyrylation

Beta-hydroxybutyrate (BHB) is a ketone body generated during fasting, ketogenic diets, prolonged exercise, and metabolic stress [30]. Beyond its role as an alternative energy substrate, BHB functions as a signaling metabolite capable of reshaping chromatin [48]. Histone lysine β-hydroxybutyrylation (Kbhb) was identified as a starvation-responsive histone modification enriched at actively transcribed genes involved in fatty acid oxidation, ketogenesis, and stress adaptation [30,49].

The biological significance of β-hydroxybutyrylation extends beyond normal physiology [30]. Cancer cells exposed to nutrient deprivation exhibit altered Kbhb profiles that promote metabolic flexibility and survival. In the nervous system, ketone body metabolism has been proposed to exert neuroprotective effects partially through chromatin remodeling mediated by Kbhb [49,50]. Emerging evidence suggests that impaired ketone utilization in AD may contribute not only to energetic deficits but also to altered chromatin states governing neuronal plasticity and stress resistance [51].

#### 2.5.2. Histone Crotonylation

Histone crotonylation is derived from crotonyl-CoA and is enriched by active promoters and enhancers. p300 can catalyze histone crotonylation, and intracellular crotonyl-CoA availability can stimulate transcription, indicating that lipid and short-chain acyl-CoA metabolism can influence chromatin through mechanisms that are distinct from acetylation [52]. In cancer, crotonylation has been linked to transcriptional activation, cell-state plasticity, and metabolic adaptation; in the nervous system, it is increasingly studied as a regulator of neuronal gene expression and injury responses [53,54].

#### 2.5.3. Histone Lactylation

Histone lactylation represents one of the most significant recent discoveries in epigenetics [47]. Histone lactylation provides an emerging link between lactate accumulation and gene regulation. Although lactate is usually discussed in the context of glycolysis, lactate production is tightly coupled to mitochondrial substrate selection, fatty acid utilization, and redox balance [55]. One study showed that lactate can drive histone lysine lactylation and activate gene-expression programs during macrophage polarization [47]. This discovery is particularly relevant to tumors, where lactate-rich microenvironments promote immune suppression and therapy resistance, and to brain disorders, where lactate exchange among astrocytes, neurons, and microglia may influence inflammatory and repair-associated chromatin states [56,57].

#### 2.5.4. Histone Succinylation, Malonylation and Propionylation

Additional acyl modifications derived from succinyl-CoA, malonyl-CoA, and propionyl-CoA further expand the metabolic control of chromatin [45]. Histone succinylation introduces a large negatively charged group that can alter nucleosome stability and DNA-histone interactions, whereas malonylation and propionylation reflect fluctuations in intermediary metabolism and lipid biosynthetic pathways [45]. These marks are especially relevant to diseases characterized by mitochondrial dysfunction, including cancer, AD, PD, Huntington’s disease (HD), amyotrophic lateral sclerosis (ALS), and multiple sclerosis (MS) [4]. Collectively, these discoveries broaden the concept of lipid–epigenome crosstalk toward a wider acylation landscape that links lipid flux, mitochondrial metabolism, and chromatin state [58].

### 2.6. Lipid Droplets as Epigenetic Organelles

Lipid droplets (LDs) were historically viewed as inert storage depots for triglycerides and cholesteryl esters [59]. They are now recognized as dynamic metabolic organelles that participate in energy homeostasis, stress adaptation, organelle communication, immune signaling, and gene regulation [59]. Emerging evidence suggests that LDs influence chromatin through regulation of acetyl-CoA availability, sequestration of signaling molecules, control of oxidative stress, and interaction with histone biology [60,61].

#### 2.6.1. Lipid Droplets as Buffers of Nuclear Metabolism

One of the principal mechanisms by which LDs influence epigenetic regulation is by controlling intracellular metabolite flux [59]. LDs continuously exchange fatty acids with mitochondria, endoplasmic reticulum, peroxisomes, and lysosomes, thereby regulating substrate availability for beta-oxidation, phospholipid synthesis, and acetyl-CoA production [60]. Because acetyl-CoA is the donor for histone acetylation, changes in LD biogenesis, lipolysis, and lipophagy can indirectly influence nuclear acetyl-CoA pools and histone acetylation. Under nutrient excess, LDs prevent lipotoxic accumulation of free fatty acids; during starvation or stress, they release fatty acids that can support mitochondrial oxidation and acyl-CoA production [60].

#### 2.6.2. Lipid Droplets and Histone Regulation

A particularly important observation is that LDs can interact with histones. In Drosophila embryos, LDs bind excess histones and help maintain histone availability for chromatin assembly [61]. Although the extent of this mechanism in adult mammalian disease remains to be fully elucidated, it supports an emerging view that LDs can shape epigenomic regulation through the control of both metabolite availability and chromatin-associated proteins.

#### 2.6.3. Lipid Droplets in Cancer and Neurodegeneration

Many aggressive tumors accumulate LDs, including glioblastoma, hepatocellular carcinoma, pancreatic cancer, ovarian cancer, prostate cancer, and breast cancer [44]. LD-derived fatty acids can support acetyl-CoA generation, ferroptosis resistance, inflammatory lipid-mediator production, and transcriptional programs linked to stemness, metastasis, and therapy resistance [62,63]. In the aging and diseased brain, LD accumulation occurs mainly in glia and is associated with mitochondrial dysfunction, lysosomal stress, inflammatory persistence, and impaired lipid trafficking [64]. APOE4-linked LD accumulation in microglia is especially important in AD because it links lipid storage to defective phagocytosis and maladaptive inflammatory gene regulation [11].

#### 2.6.4. Therapeutic Implications

The recognition of LDs as regulators of metabolism and chromatin creates new therapeutic possibilities [59]. A comparative overview of the major lipid-derived metabolites, their epigenetic targets, and disease-specific implications is presented in Table 1.

In cancer, disrupting LD-mediated metabolic buffering may reduce tumor adaptability and treatment resistance [62]. In neurodegenerative disease, restoring LD turnover and lipid trafficking may help normalize inflammatory chromatin programs in glia [64]. Future studies combining lipidomics, metabolomics, chromatin accessibility profiling, and spatial multi-omics will be essential for defining whether LDs act primarily as metabolic buffers, signaling platforms, histone regulators, or multifunctional organelles that integrate all of these processes [65].

**Table 1:** Comprehensive comparison of lipid-derived metabolites, epigenetic mechanisms, and disease-specific outcomes.

Lipid-Derived Metabolite/Pathway	Primary Source	Epigenetic Target	Cancer-Related Effects	Brain-Disorder Effects	Therapeutic Opportunities	References
Acetyl-CoA/acetate	ACLY, ACSS2, FAO	Histone acetylation	Enhancer activation, stemness, therapy resistance	Altered plasticity, inflammatory chromatin	ACLY/ACSS2/HAT modulation	[6,7,17]
SAM and choline metabolism	One-carbon and Kennedy pathways	DNA/histone methylation	Tumor suppressor silencing, methylation plasticity	Synaptic and glial methylation changes	DNMT targeting, methyl-donor balance	[23,66]
NAD+/FAO	Mitochondrial respiration	Sirtuins and deacetylation	Stress tolerance and survival	Aging, mitochondrial failure	NAD+ boosters, sirtuin modulation	[28]
BHB	Ketogenesis	HDAC inhibition, Kbhb	Stress adaptation	Neuroprotection and resilience	Ketogenic strategies	[30,48]
Crotonyl-CoA	Short-chain acyl-CoA metabolism	Histone crotonylation	Transcriptional activation, stemness	Neuronal gene regulation	Emerging chromatin targets	[52]
Lactate	Glycolysis and redox coupling	Histone lactylation	Immune escape, adaptation	Microglial and astrocyte states	Lactate/lactylation targeting	[47]
Cholesterol/oxysterols	Mevalonate pathway	Nuclear receptor chromatin programs	Membrane demand, invasion	AD, glial inflammation	LXR/PPAR modulation	[35,41]
Sphingolipids	Ceramide and lysosomal pathways	Methylation and histone marks	Apoptosis resistance	PD, ALS, MS vulnerability	Sphingolipid pathway targeting	[39,67]
Lipid droplets	Neutral lipid storage	Acetyl-CoA buffering, histone handling	Ferroptosis resistance, plasticity	APOE4 microglial lipid stress	Lipophagy and LD modulation	[60]

Collectively, these findings demonstrate that lipid metabolism influences chromatin through multiple interconnected metabolites, signaling pathways, and organelle-dependent mechanisms.

### 2.7. Bidirectional Feedback Between Metabolism and Chromatin

The relationship is not purely one-directional. In the metabolism-to-chromatin direction, changes in acetyl-CoA, SAM, NAD+, and acyl-CoA availability alter the activity of chromatin-modifying enzymes. In the chromatin-to-metabolism direction, transcriptional and epigenetic programs regulate metabolic enzymes and transporters, while histone deacetylation itself can release acetate that is recycled into acetyl-CoA. The strongest causal examples therefore combine perturbation of a metabolic node with a defined chromatin change and, ideally, rescue or reciprocal manipulation. ACLY depletion lowers histone acetylation [12]; nuclear ACSS2 can regenerate promoter-proximal acetyl-CoA [15]; p300-dependent regulation of FASN creates a chromatin-to-lipid feedback loop in prostate cancer [66]; and SOX2-mediated repression of ACSL5 reduces acetyl-CoA-consuming fatty acid synthesis and favors histone acetylation [68]. Histone acetylation can also function as a reversible acetate reservoir that contributes carbon back to acetyl-CoA metabolism [69]. These observations establish bidirectional feedback in selected systems, but the direction and strength of causality remain context-dependent and are not established for every disease association discussed in this review.

## 3. Lipid–Epigenome Crosstalk in Cancer

Cancer cells exhibit profound metabolic reprogramming, a hallmark that enables their uncontrolled proliferation, survival, and metastasis [44]. Among these metabolic shifts, alterations in lipid metabolism are particularly prominent, providing not only energy and building blocks but also crucial epigenetic regulators that drive oncogenesis [62,63]. The interplay between lipid metabolism and epigenomic remodeling in cancer often creates a permissive environment for tumor growth and progression.

An important mechanistic theme in solid tumors is that acetate and other extracellular carbon sources become epigenetically relevant under stress [70]. Low pH, hypoxia, and stromal environment can increase reliance on ACSS2-dependent acetate availability, allowing tumor cells to preserve histone acetylation even when glucose-derived citrate production is compromised [71,72]. This means that the tumor microenvironment may actively rewire which lipid-related substrates reach chromatin and thereby reshape enhancer activity, transcriptional plasticity, and treatment resistance [62].

### 3.1. Upregulated De Novo Lipogenesis and Histone Acetylation

Many cancers display upregulated de novo lipogenesis, the synthesis of fatty acids from non-lipid precursors, even when exogenous lipids are available [6,7]. ACLY occupies a metabolic branching point because citrate-derived acetyl-CoA can support both lipid synthesis and nuclear histone acetylation [16]. Importantly, increased lipogenesis does not imply that lipid synthesis itself causes a uniform surge in nuclear acetyl-CoA or global hyperacetylation. When carbon supply and ACLY/ACSS2 activity expand the accessible acetyl-CoA pool faster than biosynthetic demand, lipogenesis and histone acetylation can rise in parallel; under nutrient limitation or when fatty acid synthesis strongly consumes acetyl units, lipid anabolism can instead compete with chromatin for the shared substrate [7,16,18,68]. The dominant outcome therefore depends on substrate supply, pathway routing, subcellular enzyme localization, oxygen and nutrient status, and transcriptional demand. The SOX2-ACSL5 example discussed in Section 3.2 represents a competition-dominant state, whereas ACLY- or ACSS2-driven acetyl-CoA expansion under other conditions can support both membrane synthesis and histone acetylation.

For example, in glioblastoma (GBM), an extremely aggressive brain tumor, the deletion of CDKN2A (encoding p16) has been shown to remodel the lipidome, making GBM stem cells more susceptible to ferroptosis, a form of regulated cell death characterized by iron-dependent lipid peroxidation [73]. This lipid remodeling is intricately linked to epigenetic changes that drive tumor progression. Furthermore, under hypoxic conditions, which are common in solid tumors, cancer cells can utilize acetate as an alternative carbon source. ACSS2 converts acetate into acetyl-CoA, which can then be transported into the nucleus to support histone acetylation, promoting the expression of critical genes for adaptation to hypoxia and tumor survival [74].

These findings also suggest lipid metabolism as an upstream regulator of epigenetic heterogeneity within tumors [62]. By changing acetyl-CoA routing, redox state, and fatty acid availability, lipid pathways influence not only tumor-cell proliferation but also ferroptosis sensitivity, immune-cell function, and lineage plasticity [63]. The recent observation that ACLY inhibition can promote anti-tumor immunity underscores that metabolic-epigenetic therapy may act on cancer cells and the surrounding immune compartment, a point of major translational importance [75].

### 3.2. Fatty Acid Oxidation and Epigenetic Regulation

Another layer of complexity is that lipid signaling frequently converges on chromatin through transcription-factor complexes [44]. PPARs, LXRs, and other lipid-sensing nuclear receptors recruit coactivators and corepressors with histone acetyltransferase, deacetylase, and methyltransferase functions [33,35]. Consequently, lipid abundance can impact transcriptional regulation through receptor-guided chromatin remodeling. This receptor–chromatin interface is especially relevant in hormone-responsive cancers, metastatic adaptation, and tumor-associated stromal compartments [76,77].

While de novo lipogenesis is often upregulated, FAO also plays a complex and context-dependent role in cancer [78]. In some cancers, increased FAO provides energy and metabolic intermediates for rapid proliferation and metastasis [44]. The acetyl-CoA generated from FAO can contribute to the nuclear acetyl-CoA pool, influencing histone acetylation [44,63]. Moreover, FAO can impact the cellular NAD+/NADH ratio, thereby modulating the activity of NAD+-dependent sirtuins (HDACs) [25]. Directionally, each cycle of FAO reduces NAD+ to NADH and FAD to FADH2; if oxidative phosphorylation is intact, NADH is rapidly reoxidized, and NAD+ availability can support SIRT1/SIRT3-linked deacetylation, whereas hypoxia, electron-transport limitation, or mitochondrial damage favors NADH accumulation and lower NAD+/NADH, thereby limiting sirtuin activity [25,26]. For example, FAO-dependent cancer cells can use intact mitochondrial respiration to maintain NAD+ regeneration during nutrient stress, while neural or tumor cells in poor respiratory environment may experience reductive pressure that weakens NAD+-dependent chromatin control [26]. Changes in sirtuin activity can lead to altered histone deacetylation patterns, affecting gene expression programs in tumors that regulate cell survival, metabolism, and immune evasion [25].

In esophageal squamous cell carcinoma, SOX2 was shown to promote global histone acetylation and super-enhancer function through two linked mechanisms: increased expression of multiple histone acetyltransferases and reduced acetyl-CoA-consuming fatty acid synthesis, partly through repression of ACSL5 [68]. This provides a competition-dominant example in which lowering lipid-anabolic use of acetyl-CoA increases the fraction available for chromatin acetylation. The study combined ESCC cell models, chromatin and metabolic profiling, super-enhancer analyses, genetic manipulation of SOX2 and ACSL5, and clinical tumor correlations showing an inverse relationship between SOX2 and ACSL5 and a positive relationship between SOX2 and histone acetylation [68]. The result therefore does not contradict the parallel increase in lipogenesis and histone acetylation observed when total acetyl-CoA supply is expanded; rather, it shows that the balance can shift toward competition when substrate supply is limiting relative to pathway demand.

At the therapeutic level, brain-penetrant ACSS2 inhibitors have recently been identified in conjunction with reduced acetyl-CoA levels, lipid storage, and tumor burden in breast cancer brain metastasis models, highlighting that enzymes at the lipid–epigenome interface are druggable even in restricted disease settings [71]. More broadly, recent studies indicate that ACLY inhibition can reshape tumor transcriptional programs and antitumor immunity [71,75].

### 3.3. Lipid-Derived Signaling Molecules and DNA Methylation

Beyond direct metabolic substrates, lipid-derived signaling molecules can also influence epigenetic marks. For instance, certain fatty acids can act as ligands for nuclear receptors like peroxisome proliferator-activated receptors (PPARs), which, upon activation, recruit coactivators with HAT activity, leading to changes in gene expression [35]. Furthermore, systemic dyslipidemia, characterized by abnormal blood lipid levels, has been directly linked to epigenetic changes that drive cancer progression, suggesting a broader systemic influence of lipid metabolism on the cancer epigenome [62]. While direct links between specific lipid-derived metabolites and DNA methylation in cancer are still being actively investigated, the overall metabolic state, influenced by lipid availability, can impact the one-carbon metabolism pathway and thus SAM levels [23].

### 3.4. Cancer-Type-Specific Manifestations of Lipid–Epigenome Crosstalk

Although the core mechanisms linking lipid metabolism to chromatin regulation appear conserved across malignancies, the dominant pathways and epigenetic outcomes vary by tumor type [79,80]. Tissue origin, oncogenic drivers, hypoxia, stromal interactions, and nutrient availability shape whether tumor cells depend on fatty acid synthesis, fatty acid oxidation, acetate utilization, cholesterol metabolism, or phospholipid remodeling [79].

#### 3.4.1. Breast Cancer

Breast cancer provides an example of acetate-dependent and lipogenesis-associated chromatin remodeling. Many breast tumors show increased FASN, ACLY, ACSS2 and SREBP activity, which collectively enhance lipid synthesis and acetyl-CoA production [71,74]. Under hypoxia, ACSS2-dependent acetate availability supports histone acetylation and expression of genes involved in stress adaptation and metastatic conditions [81].

#### 3.4.2. Prostate Cancer

Prostate cancer is known to be dependent on de novo lipogenesis [70]. Increased FASN, ACLY, ACC, SCD1 and SREBP signaling supports membrane synthesis and transcriptional plasticity. p300-mediated acetylation can regulate FASN expression, creating a feedback loop in which lipid metabolism and chromatin remodeling may promote tumor growth [66]. In castration-resistant disease, increased fatty acid oxidation and cholesterol metabolism may further influence NAD+-dependent sirtuin activity and androgen-receptor-associated chromatin programs [82].

#### 3.4.3. Colorectal Cancer

Colorectal cancer illustrates how microbiome-derived lipids and short-chain fatty acids regulate chromatin [32]. Butyrate is both an energy substrate and a potent inhibitor of class I and II HDACs [32]. In normal colonocytes, butyrate is largely oxidized, whereas in colorectal cancer cells, altered metabolism can allow butyrate accumulation and HDAC inhibition, promoting differentiation, apoptosis, and growth suppression [32]. This “butyrate paradox” demonstrates that microbial metabolites can act as lipid-linked epigenetic regulators in the host [83].

#### 3.4.4. Pancreatic and Ovarian Cancer

Pancreatic ductal adenocarcinoma develops within a hypoxic and nutrient-limited microenvironment where stromal metabolites serve as important determinants [72]. Cancer-associated fibroblast-derived acetate can promote pancreatic cancer development through ACSS2-dependent metabolic rewiring and altered transcriptional programs [84]. Ovarian cancer frequently metastasizes to the lipid-rich omental niche, where adipocyte-derived fatty acids support fatty acid oxidation, acetyl-CoA generation, stemness, and chemoresistance [85]. These examples show that extracellular lipid supply and stromal metabolism can function as relevant regulators of tumor chromatin states.

## 4. Lipid–Epigenome Crosstalk in Brain Disorders

A particularly important advance is the emerging evidence that APOE4-related lipid pathology is not only metabolic but epigenetic [11]. Recent work indicates that APOE4 can drive microglial lipid dysregulation through Asxl1/LXRalpha-H3K4me3, linking defective cholesterol-handling programs to altered chromatin at lipid-responsive genes [9,11]. This is mechanistically significant because it places microglia at the center of the lipid–epigenome interface; impaired lipid efflux, lipid-droplet accumulation, lysosomal stress, and inflammation become perpetuated once encoded into chromatin states that favor maladaptive activation [64]. The evidence for this APOE4-Asxl1/LXRalpha-H3K4me3 pathway is also specified; the study combined ApoE-targeted replacement mouse models, snRNA-seq, locus-specific epigenetic assays, primary microglia cholesterol-loading experiments, and in vivo microglia-specific Asxl1 manipulation, providing mechanistic evidence with relevance to AD biology [11].

Brain disorders, particularly neurodegenerative diseases such as AD and PD, differ from cancer because they involve highly specialized, largely post-mitotic cell populations rather than clonal proliferative expansion [86]. The brain is the most lipid-rich organ in the body; thus, regulation of lipid metabolism is paramount for neuronal structure, synaptic plasticity, and overall cognitive function [87]. Dysregulation of this delicate balance not only impairs cellular integrity but also profoundly disrupts the epigenomic landscape, contributing to disease pathogenesis. Accordingly, neurodegenerative lipid–epigenome remodeling should be framed as heterogeneous, whereby hypoacetylation, hyperacetylation, altered methylation, and chromatin accessibility changes can coexist across different cell types, brain regions, and pathological stages [88,89].

### 4.1. Impaired Metabolism and Hypoacetylation in Alzheimer’s Disease

Alzheimer’s Disease is characterized by progressive cognitive decline, amyloid-beta plaques, and neurofibrillary tangles [90]. Emerging evidence points to a significant metabolic component in AD, often described as “type 3 diabetes,” where brain glucose utilization is severely impaired. The metabolic deficit also extends to lipid metabolism, particularly in microglial cholesterol handling, LD biology, and plaque-associated lysosomal lipid accumulation, as shown by APOE4- and microglia-focused studies [64,91]. In addition, mitochondrial FAO and sterol homeostasis may be altered in a cell-type- and disease-stage-dependent manner rather than uniformly across the brain [92].

In the healthy brain, while glucose is the primary energy source, astrocytes can utilize FAO to generate ketone bodies, which are then transported to neurons as an alternative fuel, especially during metabolic stress [92]. In AD, mitochondrial dysfunction can impair FAO and reduce acetyl-CoA production in vulnerable compartments [51]. This may contribute to hypoacetylation at selected plasticity- and memory-related loci, including genes such as Brain-Derived Neurotrophic Factor (BDNF) [88]. Human post-mortem data show regionally complex acetylation-pathway changes; reported marked alterations in CBP, PCAF, HDAC1, and HDAC2 in the frontal cortex, different changes in the hippocampus, and increased nuclear histone H3 acetylation mainly in the frontal cortex at end-stage disease [93]. Thus, acetylation changes in AD are best interpreted as region-, cell-type-, and stage-dependent regulatory remodeling that can include both loss of acetylation at memory-supporting loci and gain of acetylation at other neuronal or glial programs [88].

Microglia have recently been shown to selectively regulate disease-associated lipid accumulation in AD, identifying arachidonic-acid-containing bis (monoacylglycerol) phosphate as a microglia-dependent lipid signature linked to plaques, lysosomal stress, and the AD risk gene GRN [90]. In contrast, lysophosphatidylcholine and lysophosphatidylethanolamine accumulated independently of microglia and instead associated with astrocyte activation and oxidative stress. These findings are important because they emphasize a cell-specific framework beyond the current focus on abnormal lipid metabolism [64].

Cholesterol-derived oxysterols have also emerged to bridge between peripheral metabolism and brain epigenetic vulnerability [94]. One study highlighted that brain-derived 24S-hydroxycholesterol and peripherally derived 27-hydroxycholesterol can cross or signal across the blood–brain barrier and are increasingly studied as biomarkers and functional mediators in AD [94,95]. Experimental data suggest that CYP46A1-linked 24-OH may support cognition and reduce neuroinflammation, whereas elevated 27-OH is associated with synaptic dysfunction and memory loss, potentially linking systemic hypercholesterolemia to central chromatin and transcriptional disturbances [96].

For PD, recent studies further support the idea that epigenetic remodeling is not restricted to classical neuronal genes but also affects lipid-handling pathways [97,98,99]. Recent analysis reported disease-associated shifts between 5mC (5-methylcytosine) and 5hmC (5-hydroxymethylcytosine) in PD-relevant loci and identified sphingolipid metabolism-related biomarkers such as ARSB, ASAH1, GLB1, HEXB, and PSAP, suggesting that lipid catabolism, lysosomal biology, and epigenetic regulation converge in PD pathogenesis [100,101].

The gut–liver–brain connection provides bile acids, oxysterols, and microbiome-derived lipids that can shape nuclear receptor signaling, mitochondrial stress responses, and inflammatory chromatin programs in both peripheral immune cells and brain-resident glia [102]. Rather than treating these molecules as distant biomarkers, it is increasingly useful to view them as circulating chromatin-active signals that can reinforce disease trajectories over time [102,103].

Furthermore, cholesterol metabolism, which is highly compartmentalized in the brain, also intersects with the epigenome. Recent study provides a direct tauopathy-relevant example by showing that dysregulated expression of cholesterol biosynthetic genes in AD alters epigenomic signatures of hippocampal neurons [42]. In this model, cholesterol biosynthesis was severely impaired in hippocampal neurons at a late pathological stage and altered cholesterol flux was associated with hyperacetylation at neuronal genes. Functionally, activation of CBP/p300 HAT activity restored cholesterogenic gene transcription, normalized neuronal gene acetylation levels, and improved learning and memory functions even at advanced tauopathy stages [42]. This study is therefore a neurodegenerative counterpart to the cancer example of competitive acetyl-CoA allocation: lipid-anabolic flux through cholesterol biosynthesis and histone acetylation can compete for or redistribute acetyl-CoA-dependent regulatory capacity, with consequences for neuronal plasticity and cognitive resilience [42,104].

A more mechanistic comparison can be organized along three interconnected components. First is substrate directionality and heterogeneity: cancer generally amplifies the nuclear supply of acetyl donors and lipid-responsive coactivator signaling, whereas neurodegeneration more often reflects cell-type-specific failure of lipid recycling, redox buffering, and sterol homeostasis, punctuated by locus-specific hyperacetylation or inflammatory chromatin activation in particular cells and stages [79,105]. Second is cellular objective: tumor cells maintain proliferation and plasticity, while neurons and glia may be damaged when shifts toward chronic stress and inflammatory state [80,106]. Third is temporal behavior: in cancer, the lipid–epigenome axis may be adaptive and rewired, whereas in neurodegeneration, it tends to become locked into maladaptive states that are difficult to reverse [106,107]. A possible exception is the late stage of cancer, where convergent cellular failure may create a practical “point of no return” despite earlier heterogeneity.

### 4.2. Sphingolipids, Bile Acids, and DNA Methylation in Parkinson’s Disease

PD, characterized by the loss of dopaminergic neurons in the substantia nigra, also features lipid–epigenome crosstalk [108]. Recent transcriptomic and multi-omic analyses have identified specific lipid metabolism pathways, particularly those involving sphingolipids and bile acids, as key players in PD pathogenesis [100].

Sphingolipids are essential components of neuronal membranes and act as potent signaling molecules [39]. Dysregulation of sphingolipid metabolism, evidenced by altered levels of enzymes like ASAH1 and HEXB, has been identified as a biomarker for PD [100]. These lipid alterations can influence the epigenome, particularly DNA methylation. Studies have shown that perturbations in sphingolipid pathways correlate with changes in DNA methylation patterns in neurons, potentially silencing neuroprotective genes or activating pro-apoptotic pathways [97,108].

### 4.3. Impaired Cholesterol Metabolism and Epigenetic Dysfunction in Huntington’s Disease

Huntington’s disease (HD) is caused by CAG-repeat expansion in HTT, but lipid metabolic failure is now recognized as an important contributor to disease progression [109]. The brain synthesizes most of its own cholesterol, and several HD models show suppression of cholesterol biosynthetic genes and impaired sterol metabolism [110]. Mutant huntingtin also interacts with chromatin regulators such as CBP, p300 and HDAC-containing complexes, contributing to transcriptional repression and altered histone acetylation [111]. Thus, defective cholesterol synthesis and epigenetic dysfunction may reinforce each other in vulnerable striatal neurons.

### 4.4. Lipid Dysregulation and Chromatin Instability in Amyotrophic Lateral Sclerosis

Amyotrophic lateral sclerosis (ALS) is increasingly associated with lipid imbalance, mitochondrial dysfunction, and glial inflammatory remodeling [112]. Lipidomic studies have identified changes in phospholipids, sphingolipids, cholesterol esters and fatty acid metabolism in ALS models and patient biospecimens [113,114]. TDP-43 and FUS pathology also influence RNA processing and chromatin-associated regulatory programs [115]. Because mitochondrial dysfunction alters acetyl-CoA supply, NAD+/NADH balance and oxidative stress, lipid metabolic failure may contribute to epigenetic instability in motor neurons and glia [116].

### 4.5. Demyelination and Epigenetic Reprogramming in Multiple Sclerosis

Multiple sclerosis (MS) places lipid metabolism at the center of disease biology because myelin is enriched in cholesterol, sphingolipids, phospholipids, and glycolipids [117]. Demyelination disrupts lipid homeostasis and triggers metabolic adaptation in oligodendrocytes, astrocytes, microglia and infiltrating immune cells [118]. Remyelination depends on epigenetic plasticity in oligodendrocyte lineage cells, including regulation of histone acetylation, DNA methylation and chromatin accessibility [118]. Persistent inflammatory lipid mediators and oxidized lipids can impact immune activation and lesion chronicity [119].

### 4.6. Convergent and Divergent Mechanisms of Lipid–Epigenome Dysregulation Across Neurodegenerative Disorders

AD, PD, HD, ALS, and MS differ in initiating pathology but converge on mitochondrial dysfunction, impaired lipid trafficking, altered cholesterol or sphingolipid metabolism, inflammatory lipid signaling and chromatin remodeling [38,88,103,109]. The principal lipid metabolic alterations and associated epigenetic changes across major neurodegenerative disorders are summarized in Table 2.

Despite these common mechanisms, important disease-specific differences remain. AD is characterized primarily by disturbances in cholesterol trafficking, oxysterol metabolism, APOE-dependent lipid transport, and microglial lipid-droplet biology [64,126]. PD is more strongly associated with sphingolipid metabolism, lysosomal dysfunction, and bile-acid-related signaling pathways [39,108]. HD displays pronounced defects in cholesterol biosynthesis coupled with widespread transcriptional repression and histone hypoacetylation [109]. ALS is distinguished by phospholipid imbalance, mitochondrial dysfunction, and TDP-43-associated chromatin remodeling, whereas MS combines lipid-dependent neurodegeneration with immune-mediated demyelination and oligodendrocyte epigenetic reprogramming [115,118].

Taken together, current evidence supports a unified model in which lipid metabolism serves as an upstream regulator of neurodegenerative epigenomes [88]. Disease-specific lipid pathways may differ, yet their ability to reshape chromatin architecture and stabilize maladaptive transcriptional programs represents a recurring theme across diverse neurological disorders. Understanding both the convergent and divergent aspects of this lipid–epigenome axis may facilitate the development of therapeutic strategies that target shared regulatory mechanisms while identifying disease-specific molecular nodes.

## 5. A Cross-Disease Perspective: Commonalities and Divergences

Lipid flux controls chromatin substrates and cofactors, with chromatin modifications that regulate enzyme gene expression. In cancer, acetate-derived acetyl-CoA increases histone acetylation at ACACA and FASN promoters, enhancing lipogenesis and reinforcing adaptation under hypoxia [7,18]. In brain disease, maladaptive glial inflammatory states or neuronal maintenance may occur when lipid-clearance and chromatin-resilience programs fail [42].

**Figure 2:**
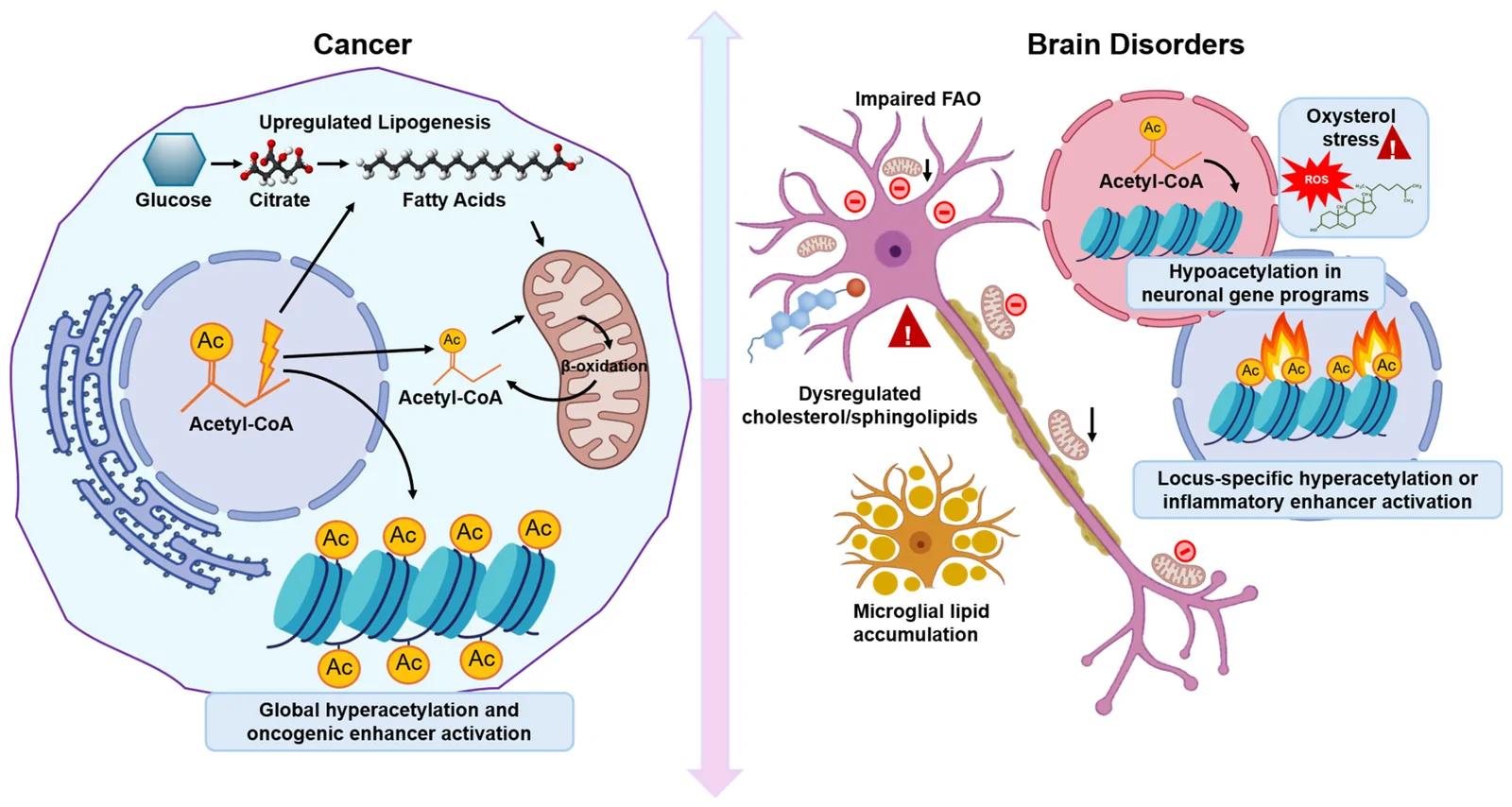
**Cross-Disease Perspective: Lipid–Epigenome Dysregulation in Cancer vs. Brain Disorders.** A comparative overview of lipid–epigenome dysregulation in cancer and brain disorders. In cancer, upregulated lipogenesis, and fatty acid oxidation lead to high acetyl-CoA availability. In brain disorders, the dominant pattern is more heterogeneous: impaired FAO, dysregulated cholesterol/sphingolipids, oxysterol stress, and microglial lipid accumulation may cause low acetyl-CoA and hypoacetylation in some neuronal programs, while specific neuronal or glial loci can show hyperacetylation or inflammatory enhancer activation depending on cell type and disease stage. Both disease contexts offer therapeutic opportunities by targeting metabolic enzymes or epigenetic modifiers, but intervention must be tailored to determine whether the relevant lipid–epigenome program should be suppressed, restored, or redirected.

Comparing the lipid–epigenome axis in cancer and brain disorders reveals some commonalities and divergences (Figure 2) [6,127]. In both settings, lipid metabolism influences chromatin through metabolite availability, cofactor balance, and membrane-derived signaling pathways that shape transcriptional state [41]. However, the biological consequence of this coupling differs between proliferative and degenerative diseases.

In cancer, lipid metabolic rewiring generally expands epigenetic plasticity [44,62]. Increased acetyl-CoA generation, acetate utilization, fatty-acid synthesis, and lipid scavenging can sustain chromatin states that favor oncogene expression, stemness, adaptive transcriptional reprogramming, and therapy resistance [7,63]. By contrast, in neurodegenerative disease, lipid dysregulation more often contributes to regulatory instability and functional decline [128]. Impaired cholesterol handling, sphingolipid imbalance, oxysterol stress, and defective lipid trafficking can restrict chromatin adaptability, amplify inflammatory signaling, and compromise neuronal maintenance programs [37,94]. Nevertheless, the neurodegenerative arm of this comparison is not a single hypoacetylated state: disease progression can generate mixed chromatin outcomes across neurons, astrocytes, microglia, and oligodendrocytes, including compensatory or maladaptive hyperacetylation at selected loci [88].

A second key difference lies in substrate economy. Tumors frequently create or access excess pools of chromatin-relevant metabolites, whereas degenerating neural systems often experience compartment-specific insufficiency, misallocation, or toxic accumulation [9,41,129]. In both diseases, lipid dysregulation becomes especially consequential when it is translated into persistent chromatin states rather than remaining a transient metabolic disturbance [90]. This common dependence makes the lipid–epigenome axis a useful comparative framework for understanding how metabolism becomes embedded within disease-specific gene-control programs.

## 6. Resolving Regulatory Heterogeneity in Lipid–Epigenome Coupling in Cancer and Neurodegenerative Disease

A major conceptual advance in this field is the recognition that lipid–epigenome coupling cannot be interpreted adequately from bulk measurements alone [65,130,131]. The same aggregate lipid or chromatin signature may arise from different cellular sources and may induce distinctive regulatory pathways depending on cell identity, anatomical location, and local nutrient environment [130]. Bulk lipidomic, transcriptomic, and epigenetic datasets therefore risk compressing biologically discrete states into a single average profile and obscuring the true origin and consequence of disease-associated changes [130,131].

In cancer, this limitation is relevant because metabolic plasticity and chromatin plasticity are unevenly distributed across the tumor ecosystem [107,132]. A bulk tumor signature suggestive of altered lipid use or acetylation potential cannot reveal whether the relevant regulatory program resides in proliferative tumor cells, invasive edge populations, therapy-tolerant persisters, or stromal and immune compartments [132]. Higher-resolution analyses, therefore, shift the key question from whether lipid metabolism is altered to which cellular states convert lipid availability into a durable regulatory advantage [80]. In this framework, lipid metabolism becomes a determinant of enhancer usage, lineage instability, and niche-specific transcriptional adaptability.

In neurodegenerative disease, the need for resolution reflects selective vulnerability [88,131]. Region-specific and cell-type-specific lipid-associated chromatin changes may emerge long before overt degeneration becomes visible at the tissue level [133]. Similar metabolic disturbances may therefore produce different outcomes in neurons, microglia, astrocytes, or oligodendrocytes, depending on each cell type’s regulatory demands and metabolic flexibility [133]. Disease progression must be incorporated into this framework because the same cell type can move from adaptive lipid buffering to maladaptive inflammatory or synaptic chromatin remodeling and finally to late-stage failure in which several regulatory programs converge toward irreversible dysfunction [88,134].

This shift also strengthens the genomic relevance of the field [135]. When lipid-associated states are examined together with chromatin accessibility, enhancer activity, transcription factor occupancy, and multiomic cell-state data, metabolism can be understood as a determinant of regulatory architecture [107,132]. From this perspective, lipid–epigenome coupling becomes a problem of regulatory heterogeneity: the biological meaning of a lipid state depends on the chromatin context in which it is embedded and the cellular program it helps stabilize [79,107]. Collectively, these findings underscore the expanding therapeutic landscape targeting the lipid–epigenome axis and highlight multiple opportunities for intervention across cancer and neurodegenerative disorders (Table 3).

### Why Metabolic–Epigenetic Therapies Often Fail in Translation

Clinical status should not be equated with clinical success. Several features of metabolic–epigenetic biology narrow the translational window. First, core metabolic pathways are shared with normal tissues, so target inhibition can produce on-target systemic toxicity before the desired disease-specific effect is reached. Second, tumors and stressed neural or glial cells can reroute carbon through redundant pathways, making single-node inhibition vulnerable to metabolic plasticity and adaptive resistance [160]. Third, bulk target abundance does not establish dependency in the relevant cell type or subcellular compartment, and pharmacodynamic measurements in blood may not report target engagement in the nucleus or disease tissue. Fourth, CNS indications add blood–brain barrier permeability, active efflux, and free-brain-exposure constraints [161]. Fifth, broad epigenetic drugs such as first-generation HDAC inhibitors act on multiple histone and non-histone substrates, creating pleiotropic effects, dose-limiting toxicity, and limited efficacy in many solid-tumor settings [143]. Finally, preclinical models may not reproduce human microenvironmental, temporal, and cellular heterogeneity. These limitations help explain why strong preclinical metabolic–epigenetic effects often translate incompletely to patients and argue for biomarker-guided combinations, cell-type-selective targets, and compartment-aware delivery rather than pathway-wide monotherapy.

## 7. Conclusions and Future Directions

The lipid–epigenome axis is emerging as a central principle in disease biology rather than a secondary consequence of metabolic imbalance [6,7]. Across cancer and neurodegenerative disorders, lipid pathways influence chromatin through metabolite supply, cofactor balance, membrane-derived signaling, and cell-state-specific regulatory circuitry [41,129]. What unites these otherwise distinct diseases is not the direction of metabolic change, but the fact that lipid dysregulation becomes pathogenic when it is converted into stable transcriptional programs.

The next challenge is to move from association to causality and to apply methodological rigor to probe disease-stage-specific mechanisms [65,129]. Future work should define how lipid flux is translated into locus-specific chromatin remodeling, which of these changes are reversible, and when they become sufficiently stable to lock cells into maladaptive states. Studies that integrate lipidomics with chromatin profiling, isotope tracing, transcriptomics, and spatially resolved tissue analysis will be especially valuable because they can connect metabolite state, regulatory architecture, and cellular identity within the same biological system [61,129,130,135].

The translational implications are equally important. In cancer, therapeutic benefit may come from disrupting the metabolic support that sustains oncogenic chromatin programs [71,75]. In neurodegenerative disease, the more realistic goal may be restoration of lipid-handling and chromatin resilience in vulnerable neural and glial populations [42]. The Paiva et al. tauopathy study illustrates this possibility by showing that CBP/p300 HAT activation can restore cholesterogenic transcription, neuronal acetylation balance, and memory performance even at advanced pathological stages [42]. The same pathway may therefore require suppression in one disease context and restoration in another, underscoring the need for cell-type-aware and stage-aware intervention strategies [66].

### Limitations and Key Gaps

Several limitations temper the conclusions that can currently be drawn. First, this article is a narrative review and, despite the structured search and evidence-weighting approach described above, cannot exclude selection or publication bias. Second, the evidence base combines transformed cell lines, primary cells, organoids, genetically engineered or pharmacologic animal models, acute nutrient-stress paradigms, and human post-mortem or biospecimen studies; these systems differ in metabolic demand, disease stage, and cell composition, limiting direct cross-model and cross-disease comparison. Third, many studies measure whole-cell metabolite pools or bulk chromatin and therefore cannot establish the local nuclear substrate concentration or the cell type responsible for an observed signal. Fourth, cross-sectional associations between lipid changes and epigenetic marks do not establish directionality; stronger causal inference requires temporal profiling, stable-isotope tracing, genetic or pharmacologic perturbation, locus-specific chromatin assays, and rescue experiments [12,13,15]. Fifth, pharmacologic studies can confound the intended metabolic mechanism with off-target or pleiotropic actions. Future work should therefore prioritize compartment-resolved metabolomics, isotope tracing, CUT&Tag/ChIP-based locus mapping, single-cell and spatial multi-omics, and longitudinal perturbation-rescue designs in human-relevant models [65,129,130,131]. These approaches are needed to distinguish cause from consequence and to determine when a lipid-associated chromatin state is adaptive, pathogenic, reversible, or irreversibly fixed.

Overall, the value of this field lies in its ability to connect genome regulation, metabolic stress, and disease progression within a single mechanistic framework. As methodological resolution improves, the lipid–epigenome axis is likely to become not only a useful explanatory model, but also a practical basis for biomarker discovery and more precise therapeutic design.

## Figures and Tables

**Table 2:** Disease-specific lipid–epigenome dysregulation across major neurodegenerative disorders.

Disease	Dominant Lipid Alterations	Principal Epigenetic Alterations	Major Affected Cells	Potential Therapeutic Targets	References
Alzheimer’s disease	APOE4 lipid dysregulation, oxysterols, LDs	Histone acetylation changes, H3K4me3/LXR remodeling	Microglia, astrocytes, hippocampal neurons	LXR, CBP/p300, lipid clearance	[9,11,41,42,90,93,120,121]
Parkinson’s disease	Sphingolipid, bile acid, and lysosomal lipid dysfunction	DNA methylation/hydroxymethylation changes	Dopaminergic neurons, microglia	GBA/lysosomal and bile-acid pathways	[10,101,122]
Huntington’s disease	Reduced cholesterol biosynthesis	Histone hypoacetylation, transcriptional repression	Striatal neurons	HDAC inhibition, cholesterol restoration	[110,123,124]
ALS	Phospholipid and mitochondrial lipid stress	Chromatin instability and altered histone states	Motor neurons, astrocytes, microglia	NAD+, mitochondrial, and lipid normalization	[112,113,117]
Multiple sclerosis	Myelin lipid loss, cholesterol, and sphingolipid remodeling	Oligodendrocyte epigenetic reprogramming	Oligodendrocytes, microglia, T cells	Remyelination, HDAC, and lipid signaling	[118,119,125]

**Table 3:** Therapeutic strategies targeting the lipid–epigenome axis. Abbreviations: ACLY.

Target/Intervention	Metabolic Function	Epigenetic Consequence	Disease Application	Clinical Status	References
ACLY inhibition	Citrate-to-acetyl-CoA conversion	Reduced histone acetylation	Cancer	Preclinical/clinical cardiovascular drug repurposing	[7,16,75,136,137]
ACSS2 inhibition	Acetate utilization	Reduced acetate-derived acetylation	Breast cancer, brain metastasis, hypoxic tumors	Preclinical	[18,71,74]
FASN inhibition	Fatty acid synthesis	Reduced lipogenic transcriptional support	Breast, prostate, and solid tumors	Clinical trials	[66,70,138,139,140]
CPT1A/FAO modulation	Fatty acid oxidation	Altered NAD+/sirtuin activity	Cancer, neurodegeneration	Preclinical	[141,142,143,144]
HDAC inhibitors	Histone deacetylation	Increased acetylation	Cancer, HD, MS models	Approved/clinical	[145,146,147,148]
DNMT inhibitors	DNA methylation	Reduced aberrant methylation	Cancer	Approved	[149,150,151]
LXR/PPAR modulation	Lipid sensing	Nuclear receptor chromatin remodeling	AD, MS, cancer	Preclinical/clinical, depending on agent	[33,35,152,153]
Ketogenic interventions	BHB production	HDAC inhibition and Kbhb	AD, PD, ALS; context-dependent cancer	Clinical investigation	[30,48,49,154,155,156,157]
Lipid droplet modulation	Storage, lipolysis, lipophagy	Acetyl-CoA redistribution and inflammatory state control	Cancer, AD	Preclinical	[9,59,60,61,92]
Multi-omics-guided precision therapy	Integrated metabolic-epigenetic profiling	Personalized target selection	Cancer and brain disorders	Emerging	[65,129,130,131,158,159]

## Data Availability

No new data were created or analyzed in this study. Data sharing is not applicable to this article.

## Abbreviations

ACACA, Acetyl-CoA Carboxylase Alpha; ACC, Acetyl-CoA Carboxylase; ACLY, ATP-Citrate Lyase; ACSS2, Acetyl-CoA Synthetase 2; AD, Alzheimer’s disease; ALS, Amyotrophic Lateral Sclerosis; ApoE, Apolipoprotein E; ATP, Adenosine Triphosphate; BHB, β-Hydroxybutyrate; CBP, CREB-Binding Protein; DNMT, DNA Methyltransferase; FAO, Fatty Acid Oxidation; FASN, Fatty Acid Synthase; FXR, Farnesoid X Receptor; GBM, Glioblastoma; HAT, Histone Acetyltransferase; HDAC, Histone Deacetylase; HMT, Histone Methyltransferase; LD, Lipid Droplet; LXR, Liver X Receptor; MS, Multiple Sclerosis; NAD+, Nicotinamide Adenine Dinucleotide; PD, Parkinson’s Disease; PPAR, Peroxisome Proliferator-Activated Receptor; RXR, Retinoid X Receptor; SAH, S-Adenosylhomocysteine; SAM, S-Adenosylmethionine; SIRT, Sirtuin; snRNA-seq, Single-Nucleus RNA Sequencing; SREBP, Sterol Regulatory Element-Binding Protein.
